# High-Throughput Proteomic Analysis of Human Dermal Fibroblast Response to Different Blood Derivatives: Autologous Topical Serum Derived from Plasma Rich in Growth Factors (PRGF) versus Leukocyte- and Platelet-Rich Plasma (L-PRP)

**DOI:** 10.3390/biom12071002

**Published:** 2022-07-19

**Authors:** Eduardo Anitua, Ander Pino, Mikel Azkargorta, Felix Elortza, Roberto Prado

**Affiliations:** 1 BTI—Biotechnology Institute I MAS D, 01007 Vitoria, Spain; ander.pino@bti-implant.es (A.P.); roberto.prado@bti-implant.es (R.P.); 2 University Institute for Regenerative Medicine and Oral Implantology—UIRMI (UPV/EHU-Fundación Eduardo Anitua), 01007 Vitoria, Spain; 3 Proteomics Platform, CIC bioGUNE, CIBERehd, ProteoRed-ISCIII, Bizkaia Science and Technology Park, 48160 Derio, Spain; mazkargorta@cicbiogune.es (M.A.); felortza@cicbiogune.es (F.E.)

**Keywords:** platelet-rich plasma, PRP, plasma rich in growth factors, PRGF, topical serum, proteomics, regenerative medicine, skin

## Abstract

Platelet-rich plasma (PRP) is nowadays used in the treatment of different types of cutaneous lesions. However, different compositions can influence clinical outcomes. Among them, the inclusion of leukocytes is controversial. High-throughput proteomics techniques were used to analyze the proteins that are differentially expressed in human dermal fibroblasts (HDFs) after treatment for 24 h with two PRP types, autologous topical serum (Endoret serum—ES) derived from plasma rich in growth factors (PRGF) and leukocyte- and platelet-rich plasma (L-PRP). The identified proteins were then classified by both Gene Ontology and Ingenuity Pathway Analysis. The obtained results show that the compositions of ES and L-PRP differ in such a way that they induce different responses in HDFs. ES-treated HDFs overexpress growth factor-related proteins, leading to protein synthesis, cell proliferation and migration. By contrast, L-PRP treatment induces a response similar to that caused by proinflammatory molecules. These data could explain the contradictory clinical results obtained for the different types of PRP, especially with respect to their leukocyte contents.

## 1. Introduction

The skin performs multiple complex functions, such as providing protection against pathogens, regulating body temperature, acting as a sensitive organ, reducing the harmful effects of ultraviolet radiation, and synthesizing of vitamin D, but it is especially notable for its function of providing a separation between the external environment and the tissues beneath it. It must ensure the protection of the organism against various aggressions, including biological, chemical, and physical agents. The integrity of the dermis and epidermis plays a key role in maintaining the homeostasis of the rest of the body. However, when the skin is injured, its protective function is lost, and injuries cause new skin to grow back through a process of wound healing. Over the years, the skin gradually weakens and loses this ability [1,2,3,4].

Regenerative medicine, with its multiple and evolving technologies, is postulated as a set of minimally invasive therapies that attempt to maintain skin homeostasis by mimicking original tissues. Included among these technologies are the application of stem cells, isolated recombinant growth factors, scaffolds composed of different materials, along with various combinations of these [5,6].

Recently, platelet-rich plasma (PRP) has been used in the treatment of skin lesions, such as trauma, burns or chronic lesions [7]. PRP is used to harness components of the blood for regenerative purposes and attempts to mimic natural wound-healing processes. Thus, blood is extracted from the patient, producing an autologous PRP that is enriched in platelets, growth factors and other bioactive molecules [8]. The use of PRP in the healing of cutaneous wounds has multiple advantages, such as its autologous nature, the possibility of use at the point of care, either as an adjuvant to other processes or as a single tool, and the possibility of being applied in multiple formulations, such as liquids, clots or membranes [7]. It has also been shown in some specific applications, such as in the treatment of diabetic ulcers, to be cost-effective compared to conventional treatments [9,10].

Plasma rich in growth factors (PRGF–Endoret) technology is one of the many types of PRP, a versatile platelet concentrate that can be obtained from small volumes of blood, which is depleted of leukocytes and erythrocytes and is activated in a controlled way with calcium chloride [11,12]. In addition, PRGF can be utilized in multiple formulations, with the time and degree of activation being varied to obtain injectable formulations that polymerize in situ in target tissues, clots, membranes, and supernatants free of fibrin and rich in growth factors [13]. Specifically, for the treatment of skin lesions, an autologous topical serum has been developed, called Endoret serum (ES), which can also be stored for long-term patient application [14,15]. ES is a viscous formulation that is enriched in growth factors and other bioactive molecules, which can be easily handled and used daily in a home-care setting without the need for repeated blood draws or specialized care, as it is stored in the patient’s own refrigerator. Previous in vitro studies have shown that ES has regenerative capacities; it was observed to stimulate dermal fibroblast activity and the synthesis of collagen and hyaluronic acid, two key components in wound healing [15]. Recently, it has also been shown in both human dermal fibroblast and 3D-organotypic ulcerated skin models that ES fosters dermal fibroblast migration, wound edge contraction and extracellular matrix synthesis [14].

However, in the area of PRP there is some controversy, which is also widespread in the area of dermatology, regarding whether the inclusion of leukocytes in PRP (L-PRP) has any positive role and even whether it could be detrimental [16,17,18]. We consider that it is key to determine whether skin cells respond in the same way to different PRP formulations. Therefore, the aim of this article is to analyze the proteins that are differentially expressed in dermal fibroblasts after treatment for 24 h with two hematological derivatives, ES and L-PRP. In this way, we intend to unravel the molecular mechanisms responsible for the therapeutic efficacy and to try to clarify whether the compositions of both types of PRP could lead to different clinical outcomes.

## 2. Materials and Methods

### 2.1. Preparation and Characterization of Blood Derivatives

Blood from three healthy donors was withdrawn into 9 mL tubes containing 0.4 mL of 3.8% (*wt*/*v*) sodium citrate as anticoagulant (BTI Biotechnology Institute, S.L., Vitoria, Álava, Spain) after obtaining written informed consent. The study was performed following the principles of the Declaration of Helsinki. Tubes were immediately centrifuged at 580 g for 8 min at room temperature in a System V centrifuge (BTI Biotechnology Institute, S.L., Vitoria, Álava, Spain).

**Figure 1:**
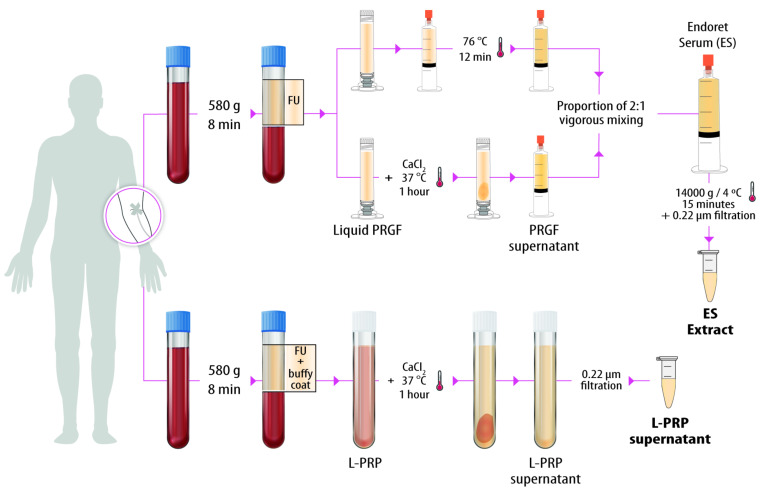
Schematic illustration of the process of obtaining the two blood derivatives. Blood was drawn from three donors in order to prepare the two blood derivatives, Endoret serum (ES) and leukocyte- and platelet-rich plasma (L-PRP). To obtain ES, the whole plasma (FU) column (excluding the buffy coat) was fractionated; a part of this PRGF was activated with 10% CaCl_2_ in order to obtain PRGF supernatant. The remaining non-activated liquid PRGF was subjected to heat treatment to jellify proteins. Finally, both components were mixed. In the case of L-PRP, the whole plasma column plus the buffy coat layer was collected and activated with 10% CaCl_2_. ES liquid extracts and L-PRP supernatants were filtered, aliquoted and stored at −80 °C until use. No sample pooling was performed.

Two types of blood derivatives were prepared from the same blood (Figure 1). To obtain the Endoret serum (ES), the whole plasma column was fractionated, excluding the buffy coat, in order to obtain non-activated liquid PRGF. Next, a part of this PRGF was activated with 10% CaCl_2_ (PRGF activator, BTI Biotechnology Institute, S.L., Vitoria, Álava, Spain) at a proportion of 20 µL/mL of plasma. After one hour of incubation at 37 °C, the clot was discarded and the PRGF supernatant was collected. The remaining non-activated liquid PRGF was subjected to heat treatment (76 °C for 12 min) to cause gelation of the plasma proteins. The last step was to vigorously mix both preparations in a proportion of one to two (Figure 1). Due to the high viscosity of ES, it cannot be placed in direct contact with cells, so liquid extracts were prepared by centrifugation at 14,000× *g* at 4 °C for 15 min. To obtain L-PRP, the entire plasma column plus the buffy coat was collected. Next, it was activated with CaCl_2_ in the same way as for obtaining the PRGF supernatant (Figure 1). L-PRP supernatants and ES liquid extracts were filtered by 0.22 μm PVDF filters, aliquoted and stored at −80 °C until use.

In order to characterize the products, a complete blood count with 5-part differential (Pentra ES 60, Horiba ABX SAS, Montpelier, France) was performed for the liquid PRGF and L-PRP, in both cases prior to activation with calcium chloride. Additionally, leukocyte, erythrocyte, and platelet concentration factors relative to the level of peripheral blood and platelet yield (%) were also calculated.

### 2.2. Cell Culture

Human dermal fibroblasts (HDFs) (ScienCell Research Laboratories, San Diego, CA, USA) were used to test the biological effect of the two blood derivatives. HDFs were cultured according to manufacturer’s instructions. Briefly, cells were maintained in culture until confluence in Fibroblast medium supplemented with Fibroblast Growth Supplement (FGS), 2% fetal bovine serum (FBS) and penicillin–streptomycin (ScienCell Research Laboratories, San Diego, CA, USA). Afterwards, HDFs were detached with animal-origin-free trypsin-like enzyme (TrypLE Select, Gibco-Invitrogen, Grand Island, NY, USA). Cell viability was assessed by trypan blue dye exclusion. Next, cells were seeded in 6-well plates at a density of 50 × 10^3^ cells/cm^2^ in serum-free medium supplemented with 20% (*v*/*v*) of the ES extract and L-PRP supernatant from the three donors and incubated with each treatment for 24 h.

### 2.3. Protein Extraction

After 24 h, the cell culture medium was discarded and the wells were rinsed with PBS. After that, to obtain cell proteins, 400 μL of cell lysis buffer was added to each well. This buffer was composed of 7 M urea, 2 M thiourea and 4% CHAPS. Samples were then incubated for thirty minutes at room temperature under agitation, to be further digested following the Filter-Aided Sample Preparation (FASP) protocol described by Wiśniewski in 2019 [19], with minor modifications. Trypsin was then added at a trypsin:protein ratio of 1:10, and the mix was incubated overnight at 37 °C, desiccated in a Rotational-Vacuum-Concentrator RVC2 25 concentrator (Christ, Osterode am Harz, Germany) and finally resuspended in 0.1% FA. Samples were stored at −80 °C until analysis.

### 2.4. Proteomic Analysis

Samples were submitted to LC–MS label-free analysis using a hybrid trapped ion mobility spectrometry–quadrupole time-of-flight mass spectrometer (timsTOF Pro with PASEF, Bruker Daltonics) coupled online to a nanoElute liquid chromatograph (Bruker). A sample of 200 ng was directly loaded in a 15 cm Bruker nanelute FIFTEEN C18 analytical column (Bruker) and resolved at 400 nL/min with a 30 min gradient. The column was heated to 50 °C using an oven.

Protein identification and quantification was carried out using MaxQuant software (Max-Planck Institute for Biochemistry, Martinsried, Germany) using default settings [20]. Searches were carried out against a database consisting of human protein entries (Uniprot/Swissprot), with precursor and fragment tolerances of 20 ppm and 0.05 Da. Only proteins identified with at least two peptides at False Discovery Rate (FDR) < 1% were considered for further analysis. Data (LFQ intensities) was loaded onto the Perseus platform [21] and further processed (log2 transformation and imputation) before statistical analysis with the Student’s *t*-test. A volcano plot was used to represent the upregulated, downregulated and unchanged proteins in our dataset. The volcano plot combines a measure of statistical significance from a statistical test with the magnitude of the change.

### 2.5. Functional Analysis

Gene Ontology (GO) enrichment analysis was carried out using the Database for Annotation, Visualization, and Integrated Discovery (DAVID) online tool [22,23]. DAVID is a GO Term annotation and enrichment analysis tool used to highlight the most relevant GO terms associated with a given gene list. Fisher’s exact test was used to determine whether the proportion of genes considered in certain GO terms or categories differed statistically significantly between the two sets of obtained data. Biological Process, Molecular Function and Cellular Component categories were assessed, and only GO Terms enriched with a *p*-value < 0.05 were considered for comparison between both blood derivatives and further discussion.

Ingenuity Pathway Analysis (IPA) (QIAGEN Digital Insights, Redwood City, California) was used for the functional analysis of the proteins identified. In this way, the calculated *p*-values determined the probability (*p* < 0.05) that the association between proteins in the dataset and a given canonical pathway, functional network or upstream regulator was explained by chance alone (Fisher’s exact test). Activation Z-score represents the bias in gene regulation that predicts whether an upstream regulator exists in an activated (positive values) or inactivated (negative values) state, based on the knowledge of the relation between the effectors and their target molecules. Only values with |Z-score| ≥ 2 were considered. With respect to the graphical representation of the data, a red/green color code was used for proteins detected in the proteomics experiment, red proteins being upregulated and green proteins downregulated. A blue/orange color code corresponds to upstream regulators or biological processes that IPA showed to be significantly related to the set of proteins under analysis, that is, with Fisher’s exact test *p* < 0.05. IPA correlates the expression pattern of the proteins under analysis with the information contained in its knowledgebase in order to predict the state of activation/inhibition of these upstream regulators/biological processes. Thus, blue suggests that the regulator/process is inhibited, whereas orange suggests that it is activated.

## 3. Results

### 3.1. Characterization of Blood Derivatives

Via hematological analysis, we performed a characterization of the plasma that originated the hematological derivatives placed in contact with the cells (Table 1). As expected, it was observed that the PRGF that originated the ES was practically depleted of leukocytes, while they were moderately concentrated in L-PRP, especially lymphocytes. Both platelet enrichment and platelet recovery were similar in both preparations (Table 1).

### 3.2. Proteomic Characterization

**Figure 2:**
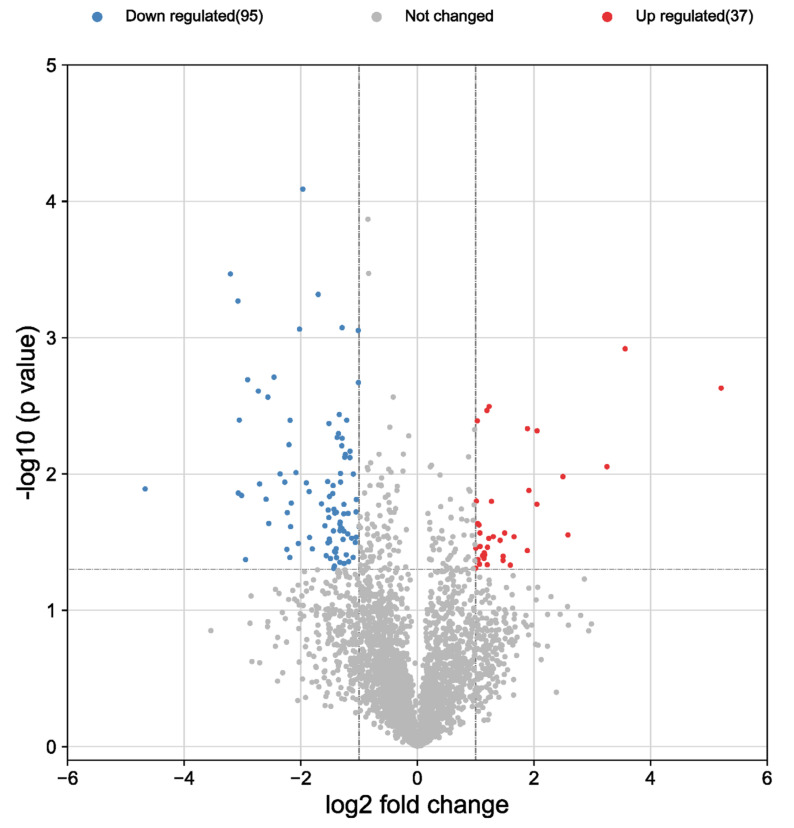
Volcano plot showing all proteins identified in the dataset. Human dermal fibroblasts (HDFs) were treated with Endoret Serum (ES) extract and with leukocyte- and platelet-rich plasma (L-PRP) supernatant. The X-axis represents the log_2_ (fold change), and the Y-axis represents the −log_10_ (*p*-value). Dashed horizontal line indicates the *p*-value cutoff (*p* < 0.05), and the two vertical dashed lines delimit downregulated (blue dots) and upregulated (red dots) proteins. The ratio is L-PRP/ES. Red dots indicate upregulated proteins in L-PRP (fold change > 2.0 and *p* < 0.05). Blue dots show downregulated proteins in L-PRP (fold change < 2.0 and *p* < 0.05). The grey dots represent proteins that do not satisfy the above conditions.

Protein extracts derived from dermal fibroblasts (HDFs) treated with both blood derivatives were subjected to analysis. In this way, differential expression of these intracellular proteins in response to treatment with ES and L-PRP obtained from three donors was observed. Overall, 3347 proteins were identified in HDFs, of which only 261 were differentially expressed as a function of treatment (Student’s *t*-test, *p* < 0.05) (Supplementary File S1). Among these 261 proteins, 95 of them were downregulated (fold change < 2.0) in L-PRP with respect to ES, whereas only 37 were upregulated (fold change > 2.0) in L-PRP compared with ES (Figure 2).

### 3.3. Functional Analysis: Gene Ontology

The first classification of the proteins differentially expressed by HDFs in response to ES and L-PRP was carried out by means of Gene Ontology (GO) analysis. A total of 114 biological processes were found to be differentially expressed between the two treatments, although statistically significant differences were found for only 58 of them (Fisher’s exact test, *p* < 0.05). Among these, we found a deregulation of processes related to tissue regeneration (wound healing, skin morphogenesis), cell adhesion (cell adhesion, cell–cell adhesion, cell adhesion mediated by integrin), extracellular matrix production (extracellular matrix organization, collagen fibril organization) and cell division (cell division, mitotic spindle organization), among others (Supplementary File S2).

### 3.4. Functional Analysis: Ingenuity Pathways

We first analyzed the canonical pathways that were differentially expressed in HDFs when treated with ES or L-PRP. Only three pathways were found to be differentially deregulated, all overexpressed in cells treated with L-PRP (Z-Score ≤ −2) compared to ES (Supplementary File S3). Of these three, the metabolism AHR signaling pathway and metabolism PXR signaling pathway were the only ones that were relevant in the context of dermal fibroblasts.

**Figure 3:**
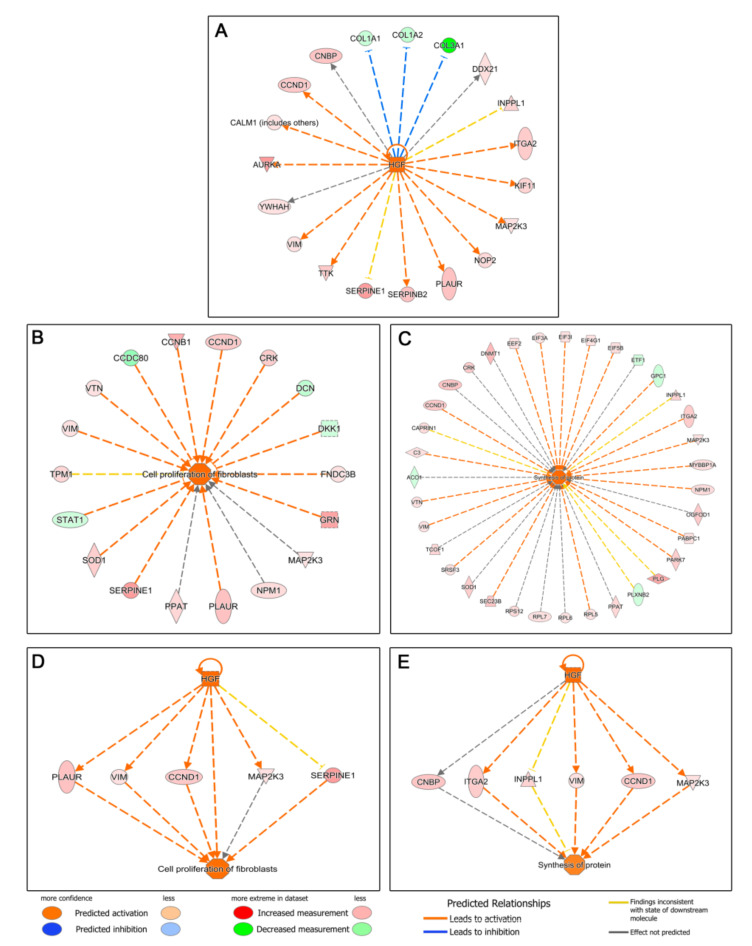
Ingenuity Pathway Analysis (IPA) of deregulated proteins related to hepatocyte growth factor (HGF) signaling in human dermal fibroblasts (HDFs) treated with Endoret serum (ES) extract. (**A**) The IPA upstream regulator analysis suggested that several HDF proteins deregulated in the ES treatment were related to the activation by HGF. IPA downstream analysis also showed that numerous deregulated proteins were associated with an induction of (**B**) fibroblast proliferation and (**C**) protein synthesis of HDFs treated with ES. Finally, when the upstream and downstream processes were correlated, certain relationships were observed for ES-treated HDFs, such as HGF with an induction of (**D**) fibroblast proliferation and (**E**) protein synthesis.

**Figure 4:**
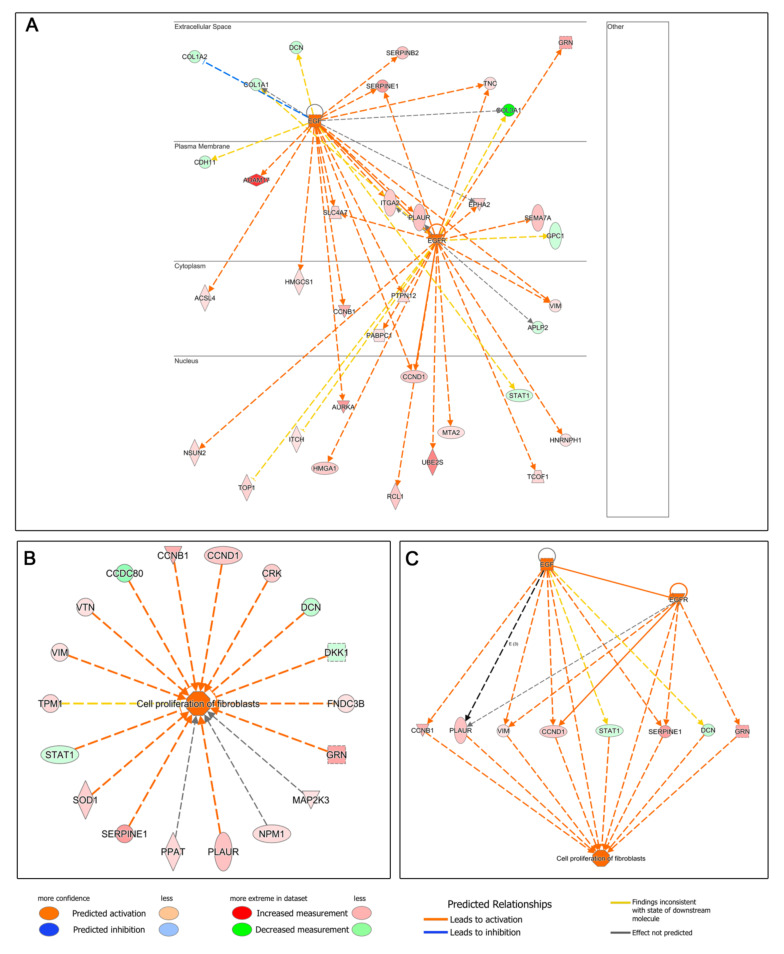
Ingenuity Pathway Analysis (IPA) of deregulated proteins related to the axis of epidermal growth factor (EGF) and EGF receptor (EGFR) in human dermal fibroblasts (HDFs) treated with Endoret serum (ES) extract. (**A**) The IPA upstream regulator analysis suggests that several HDF proteins deregulated with the ES treatment were related to the activation by EGF. (**B**) IPA downstream analysis also showed that numerous deregulated proteins were associated with an induction of the proliferation of fibroblasts. (**C**) Overall, when the upstream and downstream processes were correlated, a relationship between the EGF–EGFR axis and proliferation of HDFs treated with ES was found.

**Figure 5:**
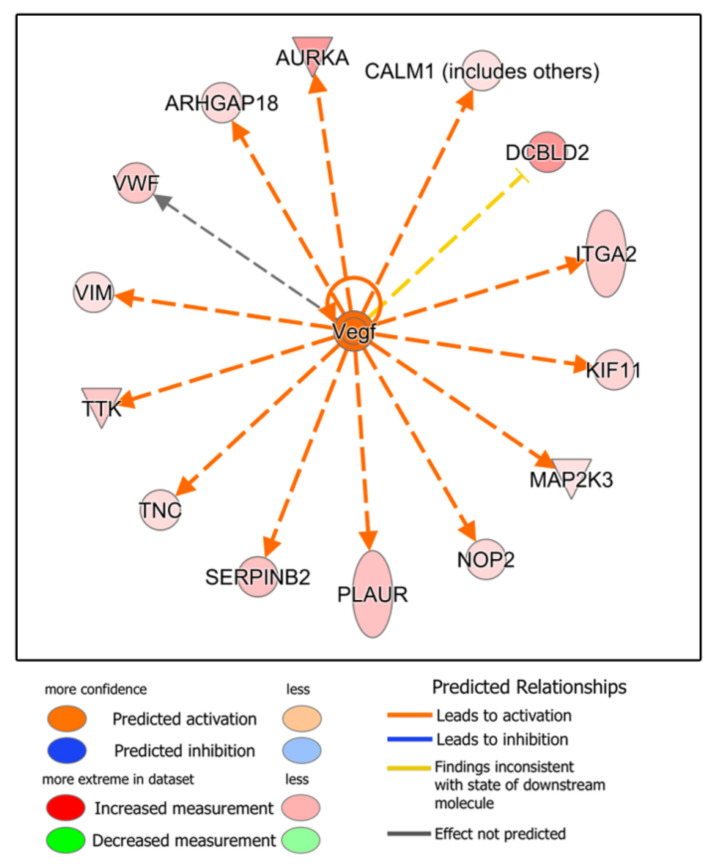
Ingenuity Pathway Analysis (IPA) of deregulated proteins related to vascular endothelial growth factor (VEGF) signaling in human dermal fibroblasts (HDFs) treated with Endoret serum (ES) extract. This figure shows that VEGF could induce the overexpression of at least fourteen proteins.

**Figure 6:**
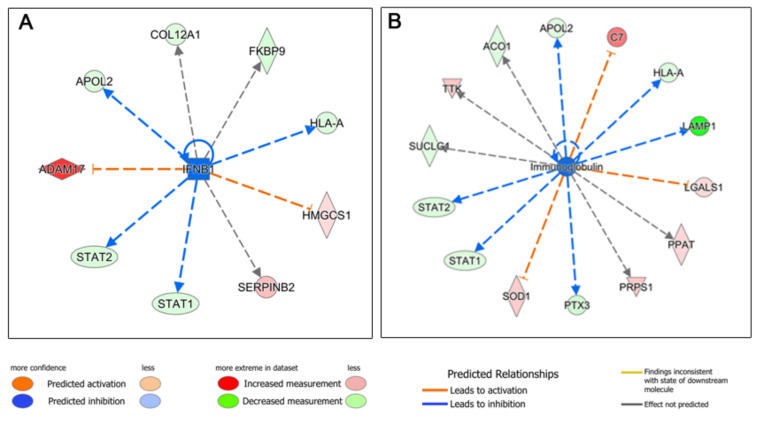
Ingenuity Pathway Analysis (IPA) of proteins deregulated in human dermal fibroblasts (HDFs) treated with leukocyte- and platelet-rich plasma (L-PRP) supernatant and related to an inflammatory response. Deregulation related to stimuli similar to those provided by (**A**) IFNB1 or (**B**) immunoglobulins was observed.

The next step was to perform an upstream regulator analysis, that is, to try to identify the cascade of upstream transcriptional regulators that may explain the gene expression changes observed when treating HDFs with the two types of blood derivatives. Seventy-three upstream regulators were identified (−2 ≤ Z-score ≥ +2), 45 of which were deregulated in favor of ES, whereas 28 were overexpressed in L-PRP-treated cells. Thus, the molecules that could be responsible for the deregulation in the expression of ES-treated HDF proteins would be, among others, members of the growth factor family and their receptors: hepatocyte growth factor (HGF) (Figure 3), epidermal growth factor (EGF) and its receptor (EGFR) (Figure 4), and vascular endothelial growth factor (VEGF) (Figure 5), among others (Supplementary File S3). In contrast, we observed that in the case of L-PRP the dysregulation could be due to inflammatory molecules, such as interferon (IFN) IFNB1, immunoglobulins (Figure 6), and IFN alpha/beta, among others (Supplementary File S3).

Once the upstream signaling processes had been identified, we focused on elucidating the functions that might be performed downstream. By analyzing Disease and Biofunctions, we expected to link the deregulated proteins we had found to specific biological functions or diseases, or to toxic processes. We only found relevant functions or processes that could be overexpressed in cells treated with ES. Thus, processes such as metabolism of protein, cell proliferation of fibroblasts, invasion of cells, synthesis of protein, cell movement, cell movement of connective tissue cells, migration of cells, or inflammatory response, among others, were identified (Supplementary File S3). When the upstream and downstream processes were correlated, certain relationships were observed for ES-treated HDFs, such as HGF with fibroblast proliferation and protein synthesis (Figure 3), or the EGF–EGFR axis with fibroblast proliferation (Figure 4), highlighting the presence of both growth factors in ES versus L-PRP.

## 4. Discussion

Through high-throughput proteomic analysis we have shown that HDFs respond differentially to treatment with ES or L-PRP. Both blood derivatives differ substantially in their preparation and their clinical application, but the major difference lies in their composition. As noted, platelet enrichment is similar; while PRGF that gives rise to ES is almost leukocyte-free, L-PRP exhibits a 1.5-fold increase in leukocytes compared with peripheral blood. The results of the PRGF hematological analyses are similar to those previously published, taking into account biological variability [24]. In the case of L-PRP, the enrichment and recovery of platelets is similar to that of PRGF, but the leukocyte differential count indicates that the lymphocytes and neutrophils in L-PRP are almost entirely absent in PRGF. We consider this aspect to be essential to explaining the results obtained.

When HDFs were treated with both preparations, a differential cellular response was observed, reflected in 3347 proteins, of which 261 were statistically significantly expressed as a function of treatment. Based on this differential expression, these proteins were catalogued and the signaling pathways in which they were involved were explored. In the case of cells treated with ES extract and compared with those treated with L-PRP, overexpression of growth factor-related pathways was found. This finding is consistent with the growth factor content of PRGF. Thus, PRGF contains HGF, EGF and VEGF—molecules that stimulate both cell proliferation and protein synthesis pathways [25]. It is evident that L-PRP also possesses growth factors, but their presence is probably counteracted by the proinflammatory environment generated by exposing HDFs to L-PRP [26].

HDFs treated with L-PRP were observed to overexpress the aryl hydrocarbon receptor (AHR) and pregnane X receptor (PXR) signaling pathways. These two pathways may have multiple functions, but it has been specifically described that the AHR has the capacity to modulate plasticity and cell differentiation and that its overactivation causes skin lesions [27]. Regarding the PXR pathway, it has recently been described to be involved in the response to cutaneous exposure to contaminants. Moreover, it has been described as a key molecule that could be involved in the development of subclinical atopic dermatitis. Thus, pollutant compounds would be able to penetrate the skin and activate PXR. Overexpression of PXR in dermal keratinocytes would drive features of atopic dermatitis, including epidermal barrier dysfunction and proinflammatory response [28,29].

There have been several studies that have evaluated the effects of leukocyte inclusion in PRGF. For example, it has been observed that the presence of leukocytes modified the morphological and biomechanical characteristics of fibrin networks by de-structuring them. In addition, the biological properties of PRGF under physiological and inflammatory conditions were also altered, since the release of pro-inflammatory cytokines (IL-1β, IL-6, IL-8 and TNF-α) from L-PRP was significantly increased under inflammatory conditions when leukocytes were included in the PRGF [30]. Another study determined the release kinetics of different growth factors in both the PRGF and L-PRP scaffolds over eight days. The results showed that PRGF fibrin matrices were homogeneous, compact and acellular, while those containing leukocytes were heterogeneous, loose and cellular. The incorporation of leukocytes resulted in a significant increase in the content of the pro-inflammatory cytokines IL-1 and IL-16, but not in the release of platelet-derived growth factors [26]. More recently, and in line with previous research, it has also been shown that the inclusion of leukocytes in PRGF scaffolds reduced their stability and reduced cell remodelling of scaffolds seeded with gingival fibroblasts, alveolar osteoblasts and human umbilical vein endothelial cells (HUVECs) [31].

Another point that is claimed for L-PRP is that the presence of leukocytes in PRP confers an additional bactericidal potential compared to PRP that does not contain them. However, it has already been shown that the inclusion of leukocytes in PRGF, similar to L-PRP, does not provide a bactericidal effect [32]. Other studies comparing the antimicrobial effect of leukocytes in PRP concluded that their presence does not provide any substantial improvement in its properties, as it is postulated that the main antibacterial effect is due to plasma and platelet microbicidal molecules [33,34].

An important limitation of this study is that we did not use a commercial kit for the preparation of L-PRP. In addition, the kits currently on the market concentrate more leukocytes than the 1.5-fold increase with respect to peripheral blood that we performed [35,36]. However, it would be expected that a higher concentration of leukocytes would be indicative of a greater inflammatory response than the one observed. Another limitation of this study would be the sample size. Human plasma has high biological variability that sometimes cannot be unraveled with small sample sizes (N = 3 donors) such as the one used in the present study. Nevertheless, the study was in line with previously published high-throughput proteomics studies with PRGF [37,38,39] and other blood derivatives [40,41]. Finally, although the ES extract was similar to the PRGF supernatant, it is not possible to rule out a possible contribution of heat-jellified proteins in the composition of the ES extract.

## 5. Conclusions

In summary, using high-throughput proteomic techniques, we have shown that the compositions of ES and L-PRP differ in such a way that they induce different responses in dermal fibroblasts after 24 h of incubation. ES-treated HDFs overexpress growth factor-related proteins, leading to protein synthesis, cell proliferation and migration. On the other hand, L-PRP treatment induces a response similar to that caused by proinflammatory molecules, such as INFB1 or immunoglobulins. Taking these data together, it would be expected that treatment with both formulations would lead to different clinical outcomes. More translational research is needed to corroborate these in vitro data.

## Figures and Tables

**Table 1:** Characterization of whole blood, PRGF and L-PRP samples included in this study. Leukocyte, platelet and erythrocyte concentrations were measured in whole blood and PRGF. Leukocyte, erythrocyte, and platelet concentration factors relative to the level of peripheral blood and platelet yield (%) are also indicated. Data are expressed as means ± SDs, *n* = 3 (biological replicates). n.d., not determined.

	Whole Blood	PRGF	L-PRP
Leukocytes (×10^3^/μL)	5.65 ± 0.18	0.15 ± 0.08	8.59 ± 0.65
Lymphocytes (%)	38.1 ± 4.1	n.d.	50.3 ± 12.5
Monocytes (%)	3.6 ± 1.5	n.d.	2.1 ± 1.3
Neutrophils (%)	53.8 ± 2.6	n.d.	44.3 ± 10.0
Eosinophils (%)	3.9 ± 1.6	n.d.	2.9 ± 2.2
Basophils (%)	0.7 ± 0.1	n.d.	0.5 ± 0.1
Erythrocytes (×10^6^/μL)	5.03 ± 0.08	0.02 ± 0.02	1.69 ± 1.09
Platelets (×10^3^/μL)	215 ± 39	431 ± 133	434 ± 164
Leukocyte concentration factor	-	0.03 ± 0.01	1.52 ± 0.15
Erythrocyte concentration factor	-	0	0.34 ± 0.22
Platelet concentration factor	-	2.0 ± 0.4	2.0 ± 0.4
Platelet yield (%)	-	68.1 ± 5.9	67.5 ± 2.1

## Data Availability

All the obtained data used to support the findings of this study are available from the corresponding author upon reasonable request.

## Supplementary Materials

The following supporting information can be downloaded at: https://www.mdpi.com/article/10.3390/biom12071002/s1: Supplementary File S1, Dataset of differential protein expression of HDFs treated with both blood derivative products (ES extract and L-PRP supernatant). Differentially expressed proteins with statistically significant differences (*p* < 0.05) are shown in boldface; Supplementary File S2, Functional analysis. Gene Ontology (GO) enrichment analysis of HDFs treated with ES extract and L-PRP supernatant. Differentially expressed GO with statistically significant differences (*p* < 0.05) are shown in boldface; Supplementary File S3, Functional analysis. Ingenuity Pathway Analysis (IPA) of deregulated proteins with statistically significant differences (*p* < 0.05) obtained for HDFs treated with ES extract and L-PRP supernatant. Three analyses were conducted: Canonical Pathways, Upstream Regulators, and Disease and Biofunctions (downstream signaling).
